# Hiccups induced by aripiprazole combined with sertraline in an adolescent with olfactory reference disorder: A case report

**DOI:** 10.3389/fpsyt.2022.793716

**Published:** 2022-07-25

**Authors:** Zhe Li, Zhenzhen Xiong, Xingmei Jiang, Zhixiong Li, Yiwen Yuan, Xiao Li

**Affiliations:** ^1^Mental Health Center, West China Hospital, Sichuan University, Chengdu, China; ^2^Sichuan Clinical Medical Research Center for Mental Disorders, Chengdu, China; ^3^School of Nursing, Chengdu Medical College, Chengdu, China; ^4^The Third Department of Clinical Psychology, Karamay Municipal People's Hospital, Karamay, China; ^5^The Fourth People's Hospital of Chengdu, Chengdu, China

**Keywords:** hiccup, olfactory reference disorder, aripiprazole, sertraline, case report

## Abstract

**Background:**

Hiccup can cause significant distress to patients and affect medication compliance. Individuals with olfactory reference disorder (ORD) who might develop persistent hiccups when treated with a combination of antidepressant and antipsychotic, leading to significant distress and impairment.

**Case summary:**

We report a rare case of an adolescent with ORD who was treated with aripiprazole combined with sertraline and who began to hiccup persistently after 6 days on this treatment. He stopped hiccupping after the aripiprazole had been suspended for 12 h. After discharge, the patient continued on sertraline alone and reported no hiccupping at 1-month follow-up.

**Conclusion:**

Clinicians should consider that the combination of aripiprazole and sertraline can induce hiccups during the acute administration period in adolescents with ORD.

## Introduction

Olfactory reference disorder (ORD) is a psychiatric condition characterized by the persistent and erroneous belief that one emits an unpleasant body odor. ORD is included in the International Classification of Diseases 11th Revision (ICD-11) as a subtype of obsessive-compulsive and related disorders (1). The prevalence of ORD has not been systematically estimated (2). ORD is typically accompanied by repetitive and excessive behaviors, such as checking for body odor repeatedly, showering frequently, and avoiding social situations, leading to significant distress and impairment of quality of life (3). However, the frequency of repetitive and excessive behavior in patients with ORD remained unknown (1).

No standard treatment for ORD has been established, although selective serotonin reuptake inhibitor (SSRI) and atypical antipsychotic have been used together (4). The antidepressant sertraline selectively inhibits the reuptake of serotonin (5-hydroxytryptamine, 5-HT) by neurons in the central nervous system, and it weakly inhibits reuptake of norepinephrine and dopamine (5). Sertraline can prevent relapse in patients with obsessive-compulsive disorder (6). The combination of sertraline or other selective serotonin reuptake inhibitors with the atypical antipsychotic aripiprazole can be effective against refractory obsessive-compulsive disorder (7). Aripiprazole acts as a partial agonist at dopamine D2 and 5-HT_1A_ receptors, while it acts as a partial antagonist at the 5-HT_2A_ receptor (8). Its agonism at the 5-HT_1A_ receptor may explain how it can be effective in combination with sertraline (7).

While aripiprazole shows a favorable metabolic profile and few adverse effects, it was reported to cause persistent hiccups in an adolescent with bipolar disorder (9) and in adult patients with bipolar disorder or schizophrenia (10, 11). Hiccups are caused by involuntary, repetitive contractions of the diaphragm and the intercostal muscles as a result of sudden glottis closure (12). Hiccups can be induced by tumors in the central nervous system, inflammatory diseases, gastrointestinal disorders, and different drug therapies (13). Although the precise etiology of hiccups is unclear, neurotransmitters such as dopamine and serotonin can play an important role (14). By extension, the use of antipsychotic medications that regulate these neurotransmitters have been associated with hiccups (14).

Here we report a case in which a Chinese adolescent with ORD suffered persistent hiccups as a result of combination therapy of aripiprazole and sertraline.

## Case description

### Chief complaints

In July 2020, a 17-year-old Chinese male was brought to the mental health center of our hospital by his parents. The adolescent reported that since 2018, he had become aware of persistent, unpleasant smell from his body, leading him to spend more than 1 h per day bathing his body and feet, to change his shoes frequently and to re-wash his feet every time he went out. He wore each pair of shoes only once and would frequently ask his parents to buy him new shoes. When someone coughed or covered his or her nose around him, he suspected that it was because of his unpleasant body odor. He reported feeling distress and anxiety, and he complained of impaired social functioning. He did not want to go to school or crowded places, and he paid a lot of attention to the expressions and movements of people around him, even counting the frequency of those actions.

### History of past illness

The patient had no history of psychiatric or medical illness, family history of mental disorders, nor medication history.

### Physical examination

The patient's temperature was 36.2°C, heart rate was 80 bpm, respiratory rate was 20 breaths per minute, blood pressure was 102/80 mmHg and oxygen saturation in room air was 98%.

### Laboratory examinations

There were no signs of infection, and results were normal for all clinicopathological examinations, including blood and urine tests, blood glucose levels, as well as tests of liver, renal, and thyroid function. Results were also normal for electroencephalography, electrocardiography, transcranial Doppler ultrasonography, and head magnetic resonance imaging.

### Further diagnostic work-up

On the Yale-Brown Obsessive-Compulsive Scale (Y-BOCS), his sub-score on obsessions was 18, and his sub-score on compulsions was 16. The total score of the Y-BOCS is 34 (0–5 is no compulsive thoughts or behaviors, 6–15 is mild, 16–25 is moderate, more than 25 is very severe symptoms). He scored 25 on the 14-item Hamilton Anxiety Scale (<7 is no anxiety, more than 14 is definitely anxiety, more than 21 is obvious anxiety, and more than 29 is very severe anxiety), 10 on the 24-item Hamilton Depression Scale (<8 is no depression, more than 20 is mild to moderate depression, and more than 35 is very severe depression), and 4 on the 32-item Hypomania Checklist (0–13 is no hypomania). The patient showed no signs of hypomania.

## Final diagnosis

Based on the patient's reported symptoms and our tests, we diagnosed him with ORD in accordance with ICD-11 criteria (1).

## Treatment

**Figure 1:**
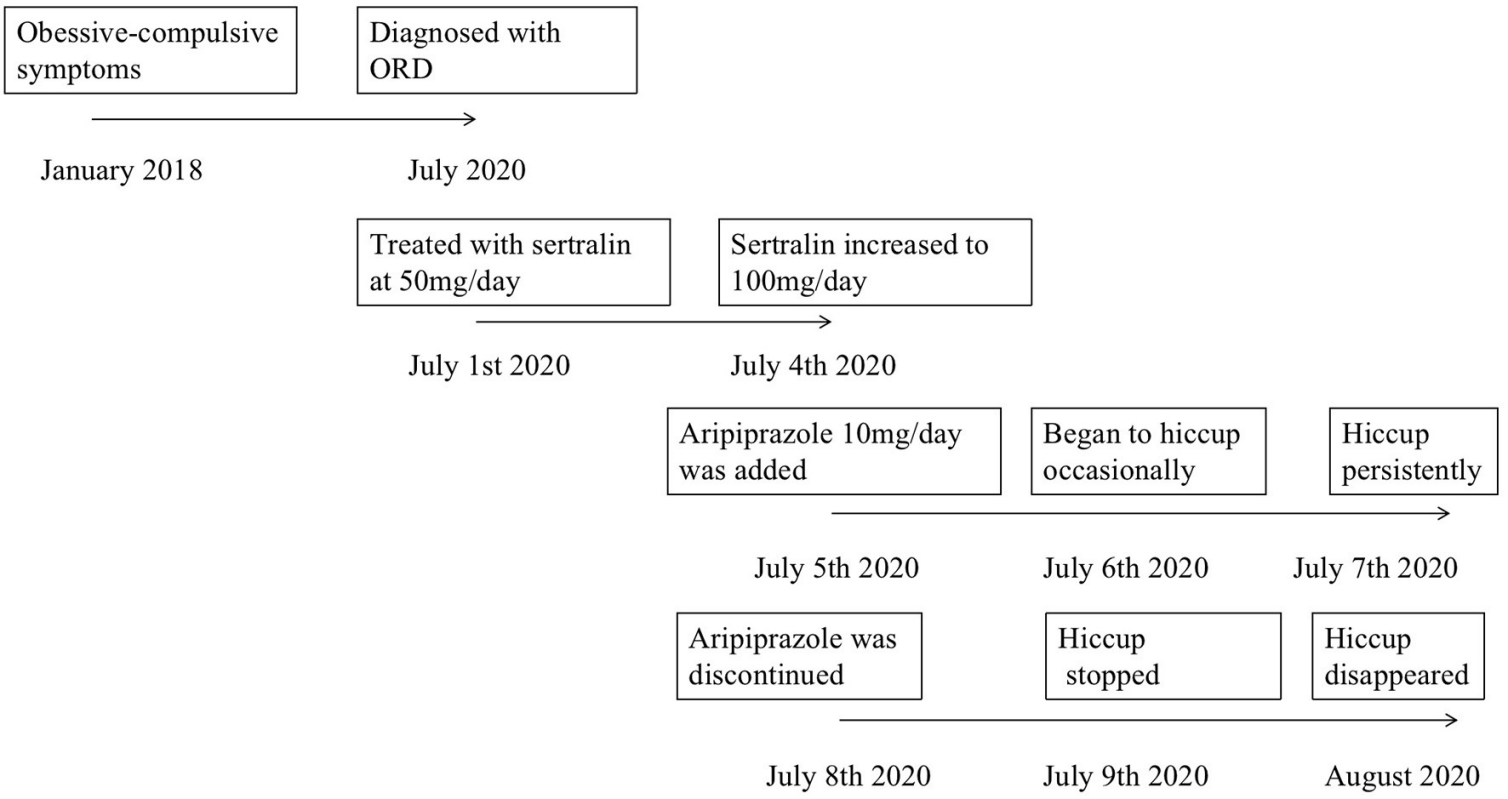
The timeline of treatment.

He was treated with sertraline hydrochloride at 50 mg/day, which was increased gradually to 100 mg/d. On day 5 of hospitalization, he reported feeling less distress and anxiety, but he still reported perceiving an unpleasant smell from his body. Therefore, aripiprazole 10 mg/d taken at night was added to his treatment. After 1 day on this combination therapy, he began to hiccup occasionally; after 2 days, the hiccup was persistent, even during the night. The patient reported being concerned that the hiccups were a side effect of the drug. After 3 days on combination therapy, aripiprazole was discontinued, and the hiccupping stopped at 12 h thereafter. After another 7 days on sertraline alone, the patient reported not perceiving any unpleasant smell from his body, and he said that he paid less attention to others' expressions and movements around him. After a total of 2 weeks' hospitalization, he was discharged on sertraline at 150 mg/d. A timeline of treatment is shown in Figure 1.

## Outcome and follow-up

The patient reported no persistent hiccupping at a follow-up visit at 1 month after discharge.

## Discussion

To our knowledge, this is the first report of an adolescent with ORD who developed persistent hiccups when treated with a combination of aripiprazole and sertraline, which then disappeared upon discontinuation of aripiprazole.

Aripiprazole may induce hiccups through multiple pathways. It binds strongly to the D3 receptor, which has been shown to play a role in hiccups (15). Aripiprazole acts as a partial agonist at the 5-HT_1A_ receptor, and its binding can lead to serotonergic facilitation of phrenic motoneuronal activity, inducing hiccups (15). Aripiprazole acts as an antagonist of 5-HT_2A_ receptors, which may also induce hiccups (16). In previous studies of patients with major depressive disorder in whom aripiprazole induced hiccupping, the hiccups stopped at 1–4 days after discontinuing the drug (17, 18), which probably reflects its half-life of 54–75 h in the blood (19). Surprisingly, our patient's hiccupping stopped just 12 h after the last drug administration, which may reflect particularly rapid metabolism or other individual factors.

Whether sertraline can induce or inhibit persistent hiccupping is unclear. It was linked to hiccupping in a patient with obsessive-compulsive disorder and attention-deficit/hyperactivity disorder (20), but it has also been proposed to suppress hiccupping through its effects on 5HT_1A_ and 5HT_2_ receptors as well as on the autonomic nervous system (21). A Previous study proposed that sertraline could reduce the binding of agonists to post-synaptic 5-HT_1A_ neuros (22). The other study found that sertraline could lead to upregulate of pre-synaptic 5-HT_1A_ receptor, leading to increased serotonin reuptake. These could be involved in the mechanism of action of the aripiprazole, which is a partial agonist of that receptor (23). Future research should examine whether and how sertraline affects risk of persistent hiccupping.

The members of the cytochrome P450 protein family mediate the metabolism of antipsychotics and antidepressants (24). This may reflect that sertraline inhibits cytochrome P450 protein CYP2D6, which degrades aripiprazole (25). Thus, the combination therapy may prolong the half-life of aripiprazole, increasing its hiccup-inducing effects on the central nervous system. Genotyping of cytochrome P450 isoenzymes may be advisable in order to avoid this adverse effect of combination therapy.

There are also some research reported that patients developed hiccup after taking aripiprazole in association with other drugs, such as benzodiazepines and methylphenidate (26, 27). The symptom improved after discontinuation one of the drugs. It is assumed that the combination of aripiprazole and other drugs may increase the risk of hiccups. In our case, the patient did not report hiccup when treated with sertraline alone, then began to hiccup persistently after treated with aripiprazole combined with sertraline. Then, hiccup subside after aripiprazole discontinuation. Therefore, the sertraline may have no contribution to the symptom. Further studies are required to clarify whether aripiprazole alone or combining with sertraline increases the risk of persistent hiccupping.

It is also possible that the our patient's persistent hiccupping reflects that men are at intrinsically higher risk of this condition (28). Hyponatremia and brain injury may also increase risk of hiccups (29, 30), but our patient did not have a history of electrolyte disturbance or brain injury.

## Conclusion

Our case suggests that physicians treating with aripiprazole in association with other drugs might expect the development of hiccup that may subside after aripiprazole discontinuation in adolescents with ORD. Genotyping of cytochrome P450 isoenzymes may be useful for detecting potential contraindications to such combination therapy. Further studies are required to clarify the role of aripiprazole alone and combined with sertraline in triggering persistent hiccupping in patients with ORD, as well as obsessive-compulsive and related disorders.

## Data availability statement

The raw data supporting the conclusions of this article will be made available by the authors, without undue reservation.

## Ethics statement

The studies involving human participants were reviewed and approved by West China Hospital Ethics Committee. Written informed consent to participate in this study was provided by the participants' legal guardian/next of kin. Written informed consent was obtained from the individual(s), and minor(s)' legal guardian/next of kin, for the publication of any potentially identifiable images or data included in this article.

## Author contributions

ZheL and ZX completed the paper and contributed equally to this work. XJ and ZhiL conducted follow-up of this patient. YY treated this patient. XL critically reviewed the diagnostic results, contributed to the preparation, and revision of the manuscript. All authors read and approved the final version of the manuscript.

## Funding

This work was partly funded by the Science and Technology project of Health Commission of Sichuan Province, 20PJ027 (ZheL), Applied Psychology Research Center of Sichuan Province, CSXL-202A08 (ZheL), Department of Human Resources and Social Security of Sichuan Province, [2020] 291-20 (ZheL), Science and Technology Bureau of Chengdu, 2021-YF05-01336-SN (ZheL), Science and Technology Department of Sichuan Province, 2022YFS0349 (ZheL), the special project of Aging Career and Industrial Research Center in 2020, and Key Research Base of Social Sciences in Sichuan Province, XJLL2020002 (ZX). The funding sources had no role in the design, execution, interpretation, analysis, or publication of the study.

## Conflict of interest

The authors declare that the research was conducted in the absence of any commercial or financial relationships that could be construed as a potential conflict of interest.

## Publisher's note

All claims expressed in this article are solely those of the authors and do not necessarily represent those of their affiliated organizations, or those of the publisher, the editors and the reviewers. Any product that may be evaluated in this article, or claim that may be made by its manufacturer, is not guaranteed or endorsed by the publisher.

## Abbreviations

ORD: olfactory reference disorder
ICD-11: the International Classification of Diseases 11th Revision
SSRI: selective serotonin reuptake inhibitor
5-HT: 5-hydroxytryptamine
Y-BOCS: the Yale-Brown Obsessive-Compulsive Scale.
